# Study of 27 aqueous humor cytokines in patients with type 2 diabetes with or without retinopathy

**Published:** 2013-08-04

**Authors:** Ning Dong, Bing Xu, Bingsong Wang, Liqun Chu

**Affiliations:** Department of Ophthalmology, Beijing Shijitan Hospital, Capital Medical University, Beijing, People’s Republic of China

## Abstract

**Purpose:**

To compare the changes in the levels of 27 aqueous humor cytokines between nondiabetic controls and patients with type 2 diabetes and to ascertain the association of these cytokines with diabetic retinopathy (DR).

**Methods:**

Undiluted aqueous humor samples were obtained from 102 nondiabetic patients (102 eyes) and 136 consecutive diabetic patients (136 eyes) who were divided into nine groups according to the Early Treatment of Diabetic Retinopathy Study severity scale. The concentrations of 27 cytokines in the aqueous humor samples were measured using a multiplex bead immunoassay.

**Results:**

Compared with the nondiabetic controls, the diabetic patients had significantly higher concentrations of interleukin-1β (IL-1β; p<0.001), IL-6 (p<0.001), IL-8 (p<0.001), monocyte chemoattractant protein-1 (p<0.001), interferon gamma-induced protein-10 (p<0.001), and vascular endothelial growth factor (p<0.001) in the aqueous humor. However, the IL-10 (p=0.002) and IL-12 (p=0.013) concentrations were significantly lower for the diabetic patients. There were no significant differences in the concentrations of other cytokines between the diabetic patients and the controls. The IL-1β, IL-6, IL-8, monocyte chemoattractant protein-1, and interferon gamma-induced protein-10 levels in the aqueous humor increased as the severity of DR increased. The correlation was significant. However, the vascular endothelial growth factor concentration was not correlated with the severity of DR. In addition, the IL-10 and IL-12 levels in the aqueous humor decreased as the severity of DR increased, and this negative correlation was significant.

**Conclusions:**

Various cytokines associated with inflammation and angiogenesis may contribute to the pathogenesis of DR, and chemokines may be more closely related to the development of this disease.

## Introduction

Diabetes mellitus (DM) has been a leading public health problem in China for the past 10 years and imposes a heavy economic burden on Chinese patients [1]. In addition, DM has become a global health problem, and its prevalence is expected to increase to 366 million worldwide by 2030 [2]. With the increasing prevalence of type 2 diabetes mellitus in the community, diabetic retinopathy–related visual impairment has become a serious public health issue; however, the pathogenesis of diabetic retinopathy (DR) is not well understood. Longer duration of diabetes, poor metabolic control, hypertension, high blood cholesterol, nephropathy, age, sex, smoking, and genetic disposition are risk factors for the development of DR, but no one has been able to fully explain the development of this diabetic complication [3]. Currently, it is known that the breakdown of the vascular barrier and inflammatory processes may play a role, and the influx of macrophages and leucocytes and inflammation are thought to be involved in the diabetic retinopathy–related damage of the eyes in patients with diabetes [4]. Previous studies have demonstrated that elevated levels of angiogenic factor, inflammatory cytokines, chemokines, and growth factors can be detected in the vitreous fluids [4-21], tear [22], and aqueous humor [23,24] of patients with DR.

Limitations of studies on aqueous humor cytokines should be noted. First, the traditional enzyme-linked immunosorbent assay method cannot detect multiple cytokines with a limited volume of aqueous humor, thus limiting the number of cytokines that can be examined [4-21]. Analyzing a greater number of cytokines would provide broader insight into the mechanisms involved. Recently, multiplex bead immunoassays have been used to detect cytokines in tears and aqueous humor because of the capacity of this type of assay to quantify multiple cytokines simultaneously in samples of small volumes [22,23,25,26]. However, the number of patients enrolled in studies using multiplex bead immunoassays was relatively small [22,23,25,26]. In addition, the process by which DM without retinopathy develops into proliferative diabetic retinopathy has a chronic course, and the change in the levels of intraocular fluid cytokines is a dynamic process. Recent studies have focused on aqueous humor cytokines in patients with severe DR or only compared cytokine levels among DR patients, diabetic patients without DR, and nondiabetic controls [6-16,23]. Only one study reported the levels of aqueous humor cytokines in patients with different DR severities that were graded according to the Early Treatment of Diabetic Retinopathy Study (ETDRS) severity scale, but the small number of patients and the limited types of examined cytokines limit the statistical power [27]. In this study, therefore, we used a multiplex bead immunoassay to compare the changes in the concentrations of 27 aqueous humor cytokines between nondiabetic controls and a large number of diabetic patients in whom DR severity was graded according to the ETDRS severity scale.

## Methods

### Subjects

Undiluted aqueous humor samples were obtained from 136 consecutive patients with type 2 diabetes (136 eyes; 71 men and 65 women) and 102 nondiabetic patients (102 eyes; 57 men and 45 women) who were undergoing cataract surgery from January 2010 to April 2012. DM was diagnosed according to the 1999 World Health Organization (WHO) criteria. Subjects had hypertension if their blood pressure was above 140/90 mmHg or they were taking any antihypertensive medications. Hypercholesterolemia was defined as fasting total plasma cholesterol above 200 mg/dl. Hypertriglyceridemia was classically defined as fasting plasma triacylglycerols (triglycerides, TG) above 200 mg/dl. Subjects were defined as nonsmokers and current smokers (nonsmokers were those who had stopped smoking for at least 1 year). The inclusion criterion for both groups was the absence of any retinal or optic nerve disease except diffuse retinopathy in the study group. The exclusion criteria included (1) any other ocular condition (e.g., glaucoma, uveitis), (2) a history of ocular surgery, and (3) a history of ocular inflammation.

This study was approved by the Ethics Committee of Beijing Shijitan Hospital, Capital Medical University, Beijing, People’s Republic of China and was performed in accordance with the Declaration of Helsinki. Informed consent was obtained from all patients before they participated in the study.

### Design and Procedure

This study was a comparative cross-sectional study and was performed at Beijing Shijitan Hospital, Capital Medical University, Beijing, People’s Republic of China.

Patients underwent a complete ophthalmologic examination and a general physical examination that included assessments of visual acuity and relative afferent pupillary defects (RAPD), an electroretinogram (ERG), slit lamp–assisted biomicroscopy of the anterior segment, a fundus examination, and fluorescence fundus angiography (FFA), which was used for the clinical diagnosis of DR. Diabetic retinopathy severity was confirmed with standardized fundus color photography and FFA within 2 days after surgery and was graded using the modified ETDRS severity scale [28,29].

At the time of cataract surgery, a limbal paracentesis was made with a sterile tuberculin syringe. Undiluted aqueous humor samples (0.1–0.2 ml) were aspirated into a syringe. The samples were immediately frozen and stored at −80 °C until analysis [23].

### Multiplex analysis of cytokines in aqueous humor samples

A Bio-Plex multiplex assay (Bio-Plex Human Cytokine 27-plex panel; Bio-Rad, Hercules, CA) was used to measure the concentrations of 27 human aqueous humor cytokines: interleukin-1β (IL-1β), IL-1rα, IL-2, IL-4, IL-5, IL-6, IL-7, IL-8, IL-9, IL-10, IL-12, IL-13, IL-15, IL-17, basic fibroblast growth factor (b-FGF), EOTAXIN, granulocyte colony-stimulating factor (G-CSF), granulocyte macrophage colony-stimulating factor (GMCSF), interferon-gamma (IFN-γ), interferon-induced protein-10 (IP-10 or CXCL10), monocyte chemotactic protein-1 (MCP-1 or CCL2), macrophage inflammatory protein-1α (MIP-1α or CCL3), macrophage inflammatory protein-1β (MIP-1β or CCL4), platelet-derived growth factor-BB (PDGF-BB), regulated upon activation normal T-cell expressed and secreted (RANTES), tumor necrosis factor-alpha (TNF-α), and vascular endothelial growth factor (VEGF). The analysis was performed according to the manufacturer’s instructions. Standard curves were generated using the Bio-PlexTM 200 System (software version 6.0; Bio-Rad Laboratories) and were used to calculate the cytokine concentrations in the aqueous humor samples.

### Statistical analysis

Data were recorded as the means±SD (standard deviation) or the median and range. The statistical analyses were performed using the program SPSS for Windows Version 17.0. The Pearson χ^2^ test was used to compare the proportions of qualitative variables. The Student *t* test and the Mann–Whitney U test were used to compare the means of the quantitative variables between two independent groups. The Kruskal–Wallis test was used to compare multiple groups. Spearman’s rank-order correlation coefficients were used to assess the relationship between the concentrations of the assayed cytokines and DR severity. A p value less than 0.05 was accepted as statistically significant.

## Results

### Characteristics of controls and patients with diabetes mellitus

Table 1 shows the demographic and clinical characteristics of the patients included in the diabetic group and the control group. All patients and controls were from the same narrow geographic area (Beijing and surrounding area) to exclude individuals who might have different conditions. The mean age of the patients with diabetes mellitus was 66.7 years (range, 56–75 years) and that of the individuals without diabetes mellitus was 69.3 years (range, 61–82 years). The mean duration of diabetes was 18.1 years (range, 1–36 years), and the mean HbA1c value was 7.8% (range, 5.5% to 10.6%).

**Table 1:** Baseline characteristics of patients with DM (n=136) and controls (n=102).

Characteristics	DM	Controls	P value
Number	136	102	-
Gender			0.573^a^
Male (%)	71 (52.2)	57 (55.9)	
Female (%)	65 (47.8)	45 (44.1)	
Age (SD)	66.7 (6.81)	69.3 (6.36)	0.159^b^
Hypertension (%)	76 (55.9)	40 (39.2)	0.011^a^
Body mass index (SD)	29.62(4.81)	27.32 (3.76)	0.061^b^
Hypercholesterolemia (%)	39 (28.7)	24 (23.5)	0.373^a^
Hypertriglyceridemia (%)	36 (26.5)	20 (19.6)	0.217^a^
Blood glucose level (SD)	8.6 (2.56)	5.2 (0.68)	<0.001^b^
Glycosylated hemoglobin(SD)	7.8 (1.84)	5.3 (0.44)	<0.001^c^
Smoking (%)	42(29.6)	25(24.5)	0.382^a^

^a^Pearson χ^2^ test. ^b^Student *t* test. ^c^Mann–Whitney U test

A history of hypertension was more frequently recorded for the diabetic patients than for the controls. A history of smoking was more common in the diabetic patients than in the controls without reaching statistical significance. There were no significant differences in cholesterol and triglycerides between the patients and the controls.

### Cytokine concentrations in aqueous humors

Table 2 shows the concentrations of the assayed cytokines. The positive detection rates were more than 80% for 22 cytokines. The positive detection rates for the other five cytokines were as follows: TNF-α (60%), IL-17 (40%), G-CSF (32%), IFN-γ (22%), and MIP-1α (20%). These five cytokines were not included in the statistical analysis because of the low detection rates.

**Table 2:** The concentrations of the assayed cytokines in the aqueous humor samples of the diabetic (n=136) and controls (n=102).

Cytokine	Patients with diabetes mellitus		Controls		P value^a^
	Median	Range	Median	Range	
IL-1β	8.0	0–104	1.0	0–38	<0.001
IL-1rα	16.2	0–336	13.5	0–216	0.556
IL-2	1.6	0–108	1.8	0–126	0.868
IL-4	1.3	0–124	1.6	0–102	1.000
IL-5	1.1	0–141	2.4	0–161	0.812
IL-6	27.5	0–365	13.5	0–76	<0.001
IL-7	4.6	0–89	2.1	0–76	0.123
IL-8	17.0	0–198	8.0	0–76	<0.001
IL-9	3.6	0–187	2.8	0–98	0.575
IL-10	5.0	0–25	6.0	0–55	0.002
IL-12	8.0	0–46	10.0	0–105	0.013
IL-13	2.6	0–36	3.5	0–28	1.000
IL-15	2.3	0–56	1.6	0–86	0.768
IL-17	-	-	-	-	-
b-FGF	12.6	0–165	9.1	0–78	0.654
Eotaxin	6.5	0–95	5.1	0–112	1.000
G-CSF	-	-	-	-	-
GM-CSF	9.7	0–86	8.5	0–64	1.000
IFN-γ	-	-	-	-	-
IP-10	4.0	0–76	1.0	0–6	<0.001
MCP-1	385.5	58–2568	70.5	7–811	<0.001
MIP-1α	-	-	-	-	-
MIP-1β	28.3	0–186	31.5	0–202	0.613
PDGF-BB	3.2	0–46	4.5	0–61	0.716
RANTES	4.6	0–76	5.2	0–67	1.000
TNF-α	-	-	-	-	-
VEGF	982.0	26–1888	66.0	11–676	<0.001

^a^Mann–Whitney U test

Compared with the nondiabetic controls, the diabetic patients had significantly higher concentrations of IL-1β (p<0.001), IL-6 (p<0.001), IL-8 (p<0.001), MCP-1 (p<0.001), IP-10 (p<0.001), and VEGF (p<0.001) in the aqueous humor samples. However, the IL-10 (p=0.002) and IL-12 (p=0.013) concentrations in the samples from the diabetic patients were significantly lower than the concentrations in the control samples. There were no significant differences in the concentrations of other cytokines between the diabetic patients and the controls.

### Association between cytokine concentrations and retinopathy severity

**Figure 1:**
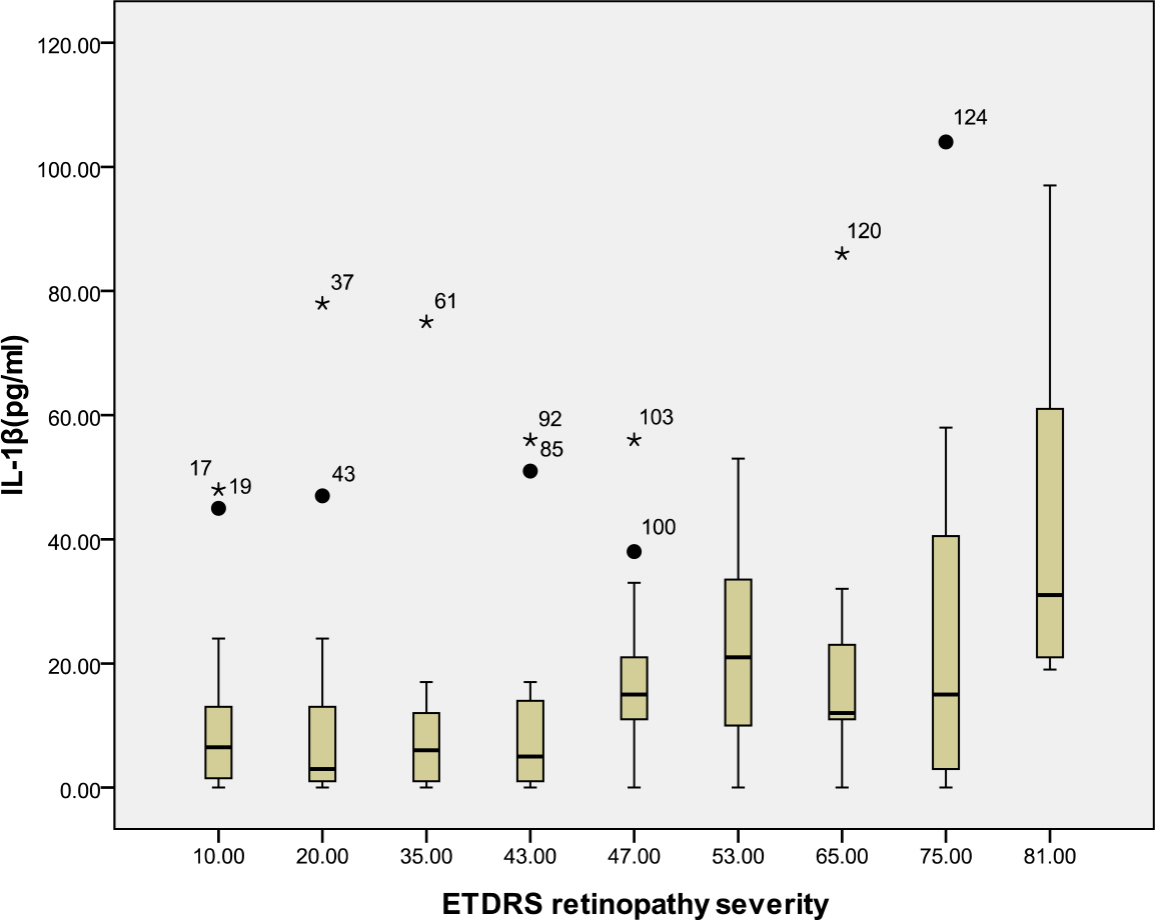
Relationship between the severity of diabetic retinopathy (DR) and the aqueous interleukin-1β level. The results by measuring cytokines in the aqueous humor samples shows that the interleukin-1β (IL-1β) level in aqueous humor increased with increasing severity of DR, and this correlation was significant (r=0.298, p<0.001). Table 3 shows the sampling sizes for each group according to the ETDRS retinopathy severity scale.

**Figure 2:**
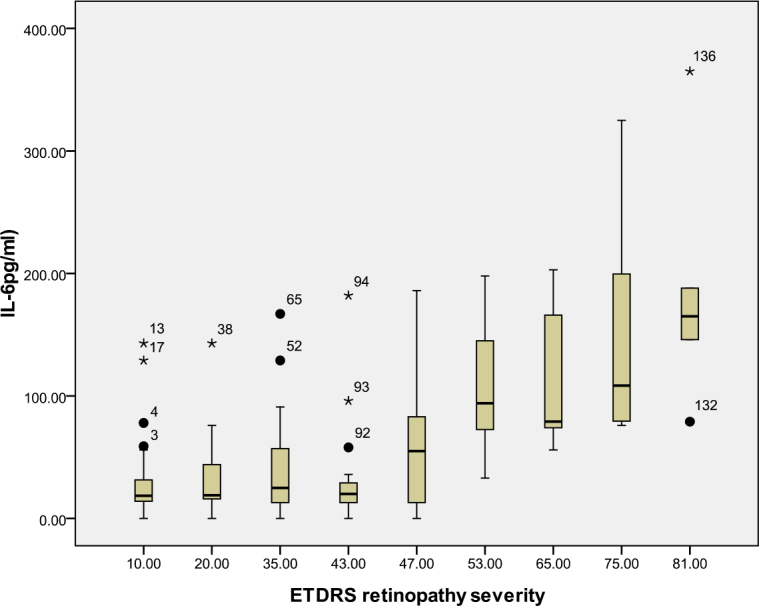
Relationship between the severity of diabetic retinopathy (DR) and the aqueous interleukin-6 (IL-6) level. The results by measuring cytokines in the aqueous humor samples shows that the IL-6 level in aqueous humor increased with increasing severity of DR, and this correlation was significant (r=0.468, p<0.001). Table 3 shows the sampling sizes for each group according to the ETDRS retinopathy severity scale.

**Figure 3:**
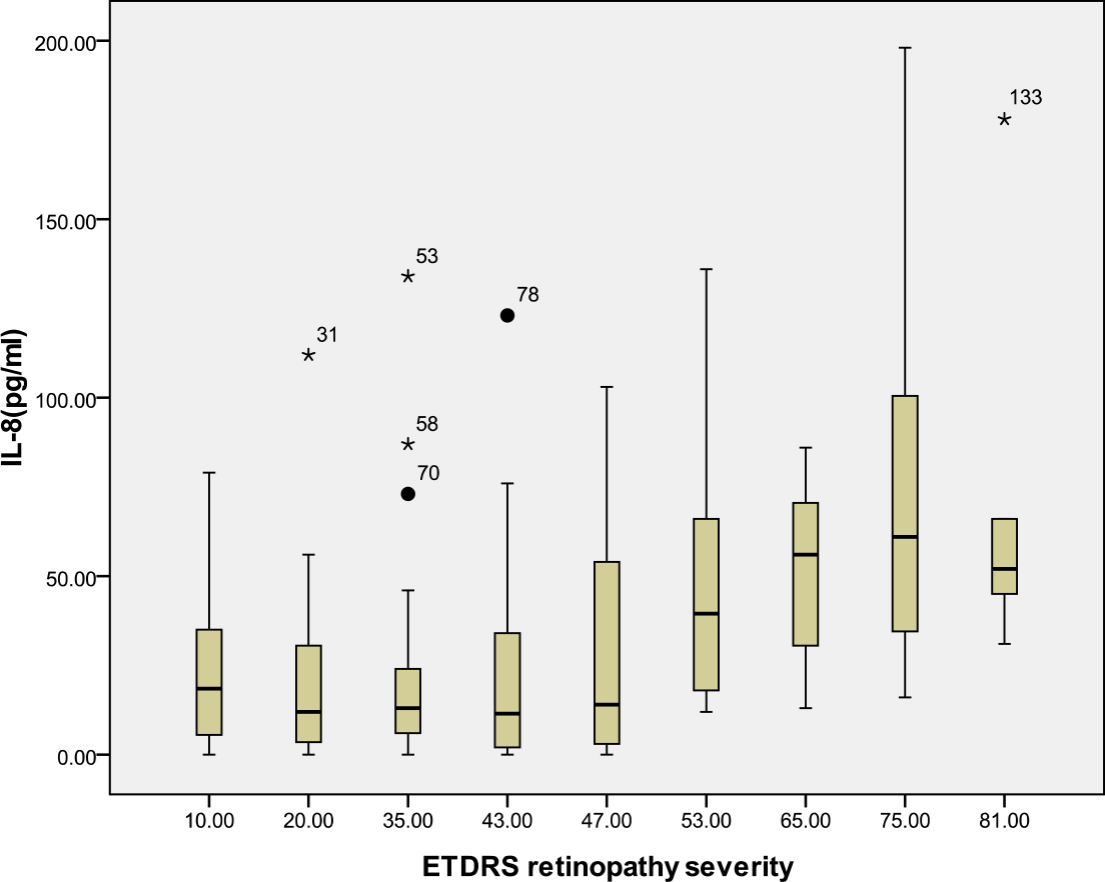
Relationship between the severity of diabetic retinopathy (DR) and the aqueous interleukin-8 (IL-8) level. The results by measuring cytokines in the aqueous humor samples shows that the IL-8 level in aqueous humor increased with increasing severity of DR, and this correlation was significant (r=0.309, p<0.001). Table 3 shows the sampling sizes for each group according to the ETDRS retinopathy severity scale.

**Figure 4:**
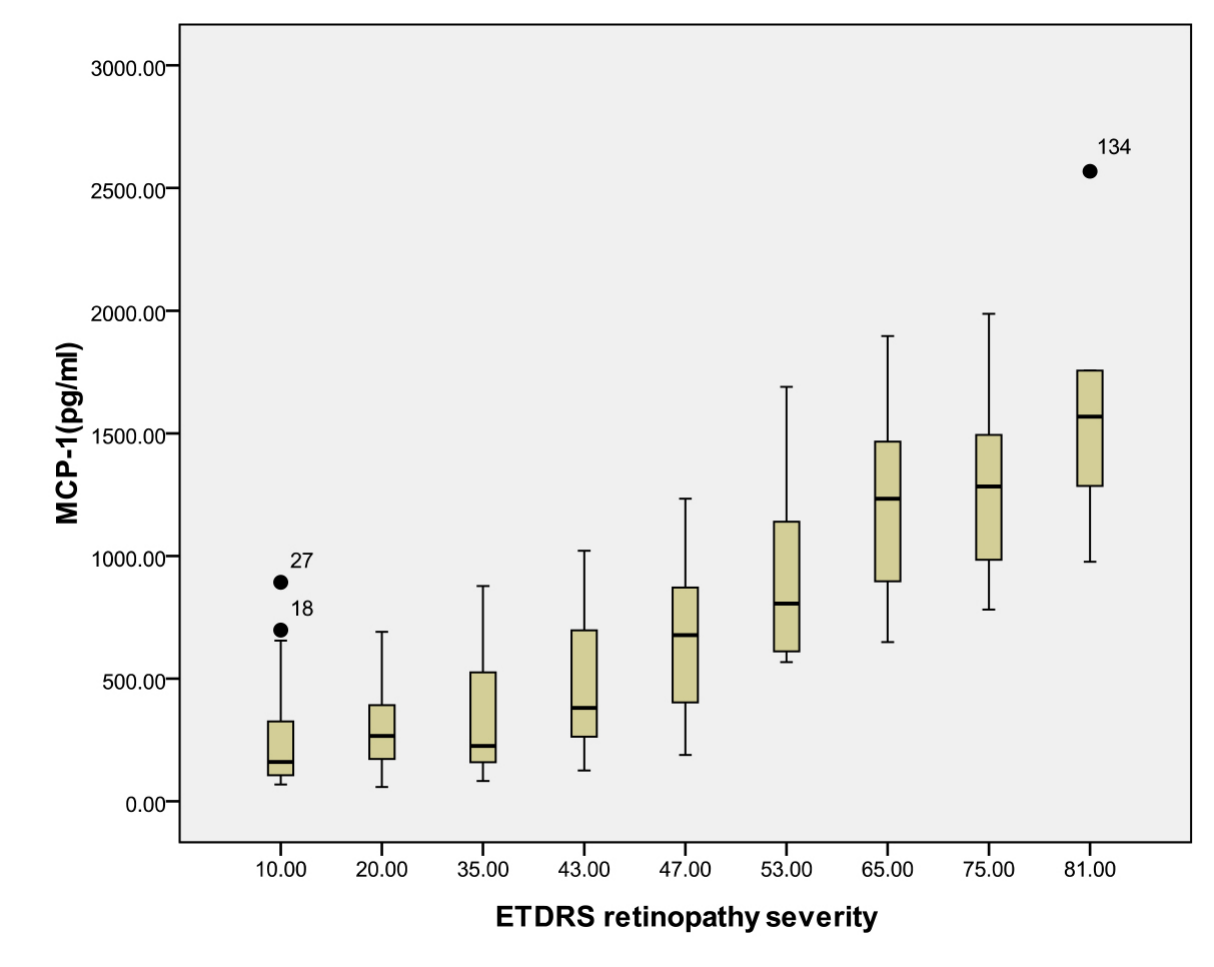
Relationship between the severity of diabetic retinopathy (DR) and the aqueous monocyte chemoattractant protein-1 (MCP-1) level. The results by measuring cytokines in the aqueous humor samples shows that the MCP-1 level in aqueous humor increased with increasing severity of DR, and this correlation was significant (r=0.693, p<0.001). Table 3 shows the sampling sizes for each group according to the ETDRS retinopathy severity scale.

**Figure 5:**
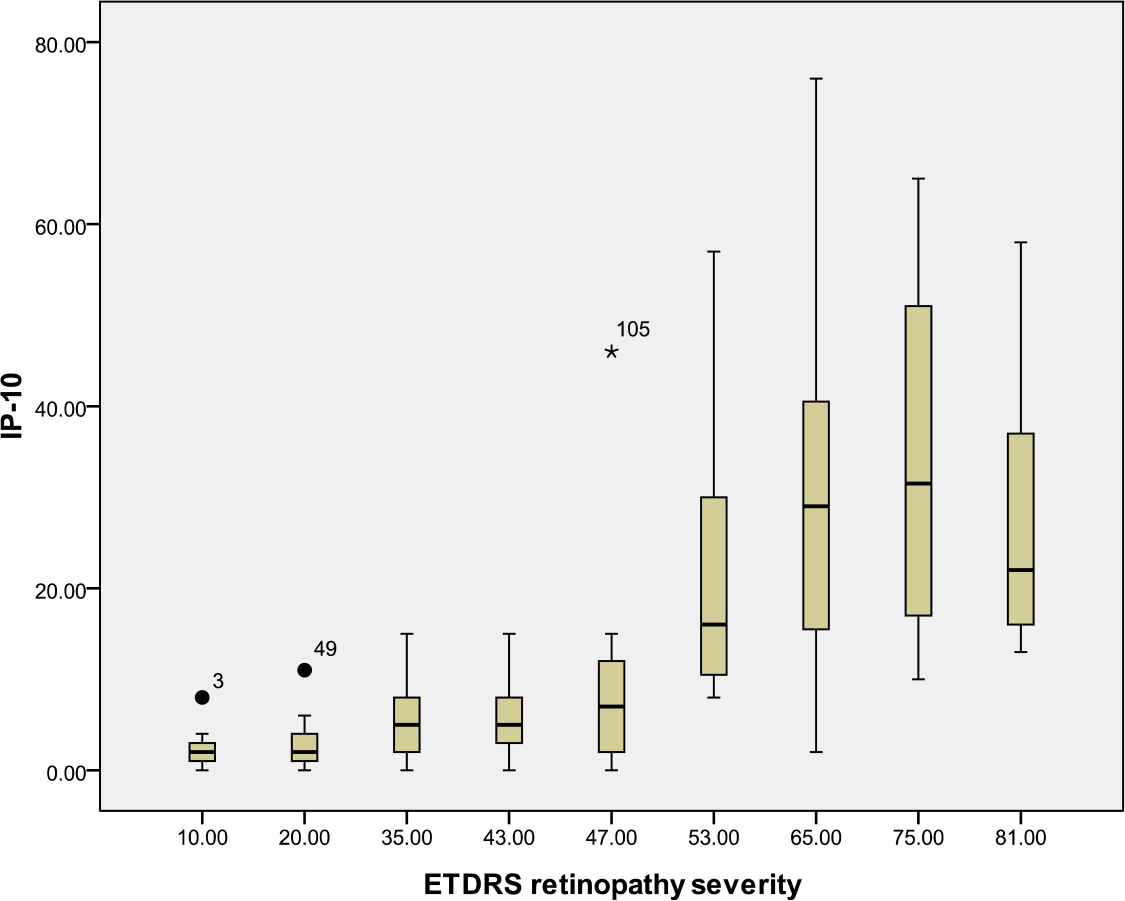
Relationship between the severity of diabetic retinopathy (DR) and the aqueous interferon gamma-induced protein-10 (IP-10) level. The results by measuring cytokines in the aqueous humor samples shows that the IP-10 level in aqueous humor increased with increasing severity of DR, and this correlation was significant (r=0.674, p<0.001). Table 3 shows the sampling sizes for each group according to the ETDRS retinopathy severity scale.

**Figure 6:**
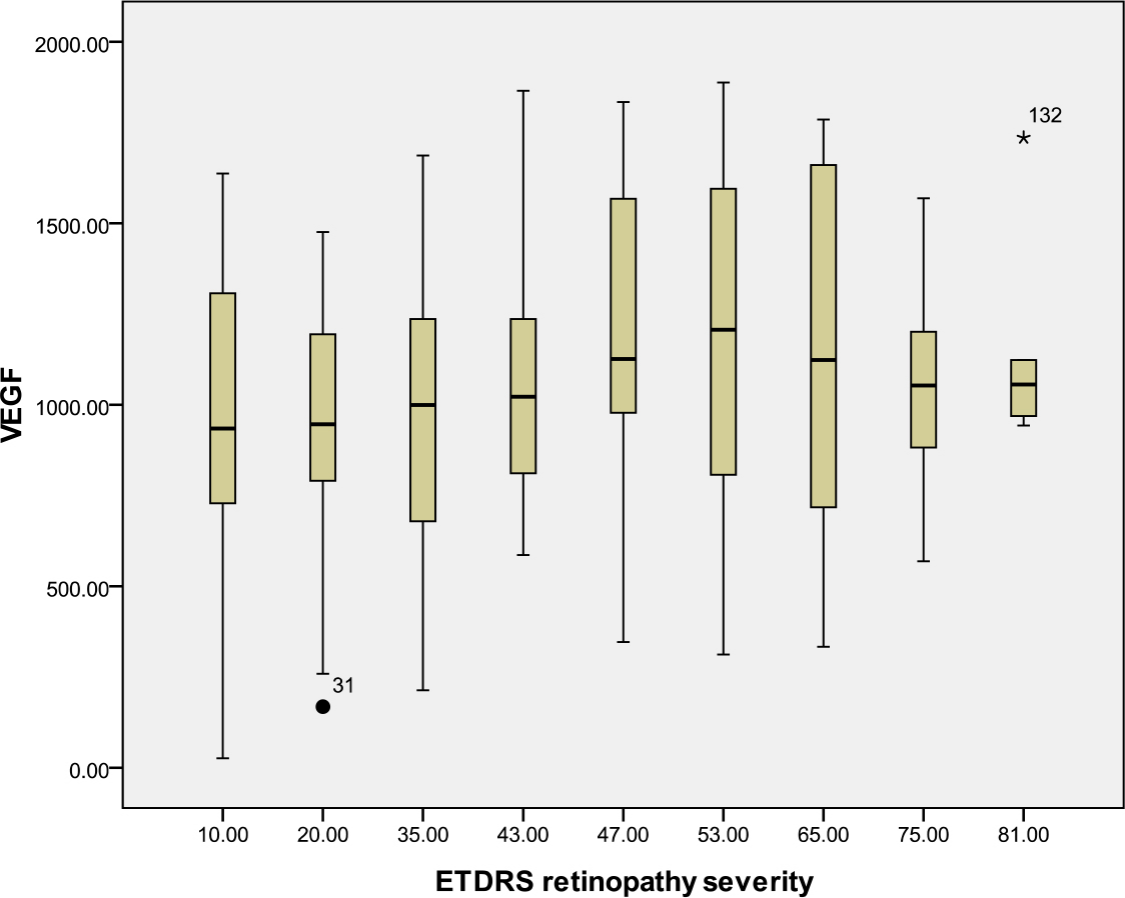
Relationship between the severity of diabetic retinopathy (DR) and the aqueous vascular endothelial growth factor (VEGF) level. The results by measuring cytokines in the aqueous humor samples shows that the VEGF level in aqueous humor did not increase with increasing severity of DR, and this correlation was not significant (r=0.161, p=0.062). Table 3 shows the sampling sizes for each group according to the ETDRS retinopathy severity scale.

**Figure 7:**
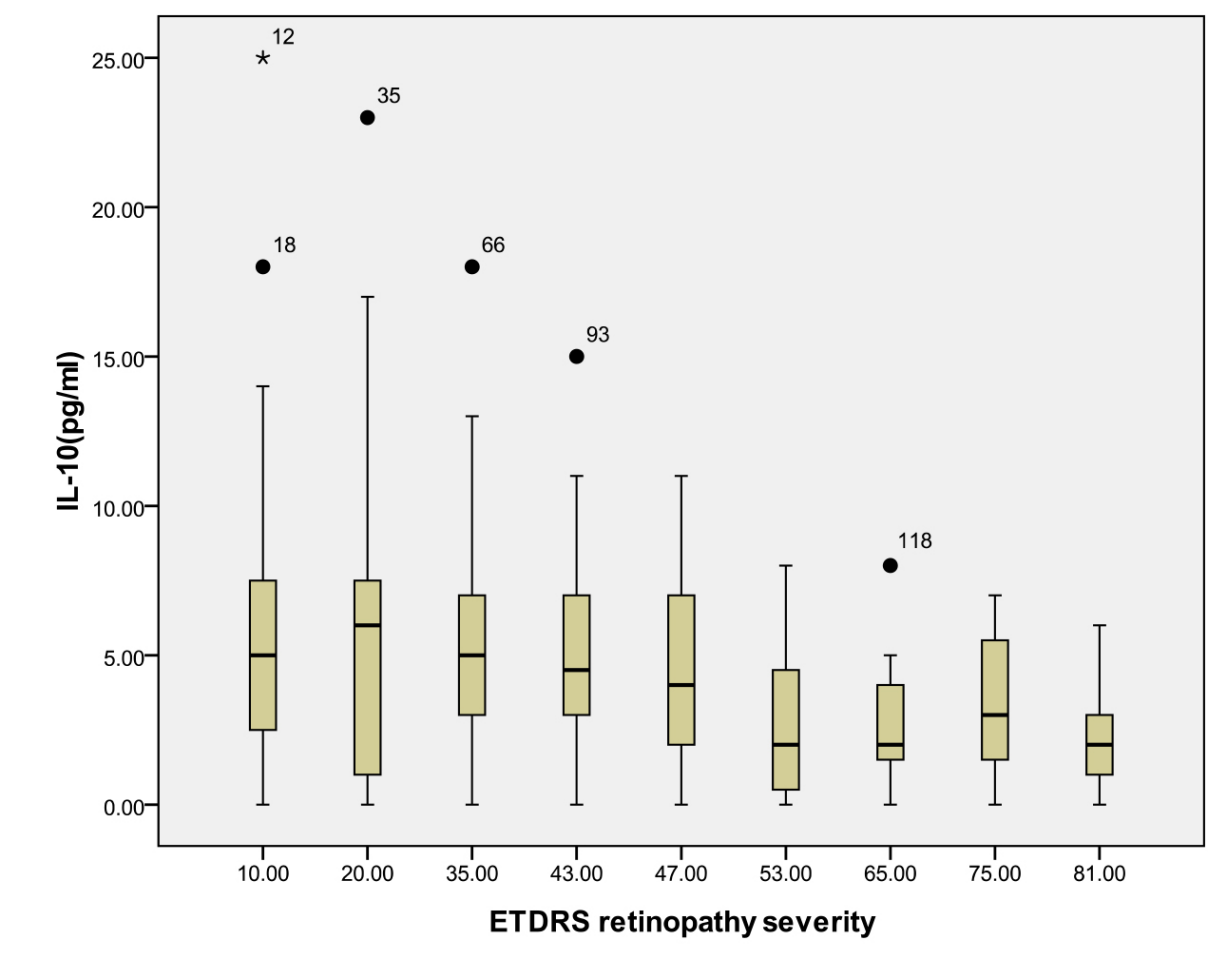
Relationship between the severity of diabetic retinopathy (DR) and the aqueous interleukin-10 (IL-10) level. The results by measuring cytokines in the aqueous humor samples shows that the IL-10 level in aqueous humor decreased with increasing severity of DR, and this correlation was significant (r=–0.206, p=0.016). Table 4 shows the sampling sizes for each group according to the ETDRS retinopathy severity scale.

**Figure 8:**
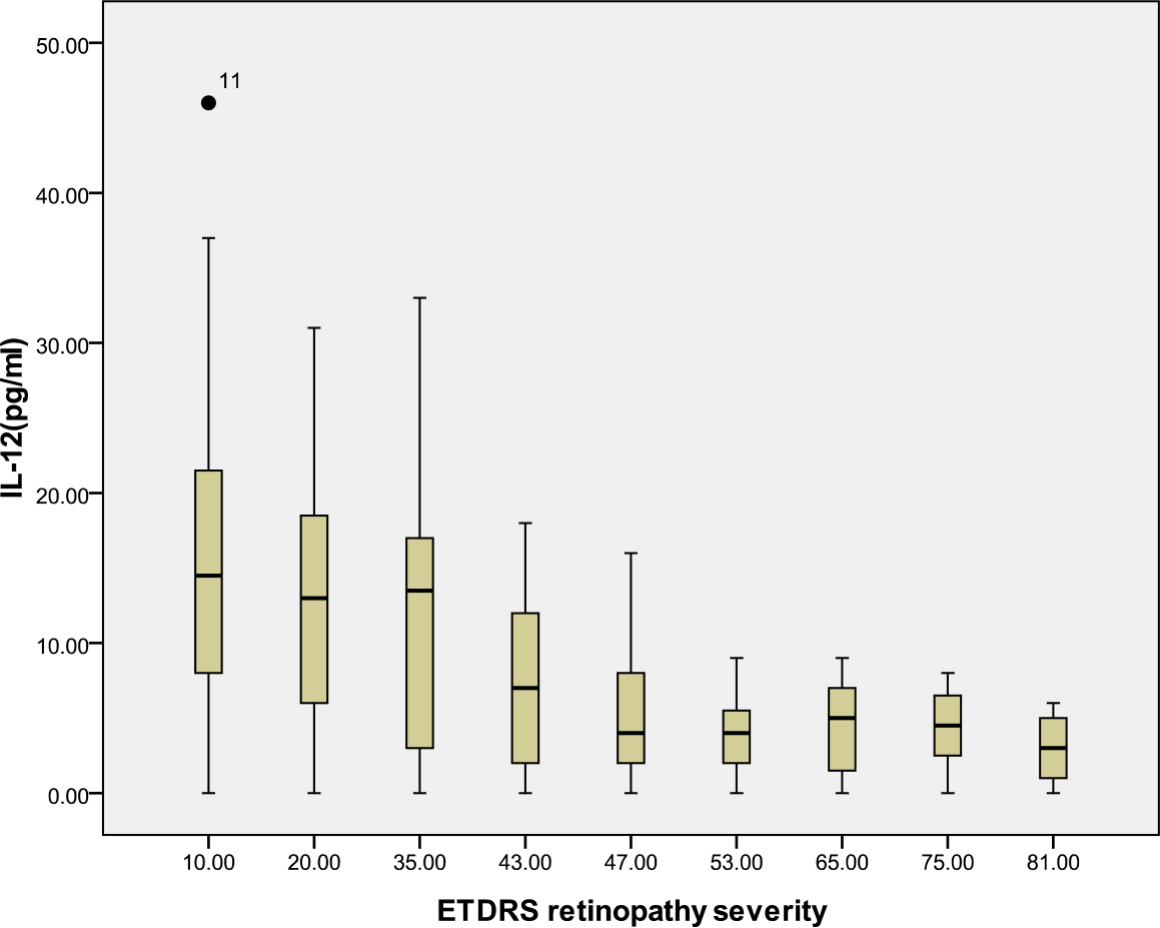
Relationship between the severity of diabetic retinopathy (DR) and the aqueous interleukin-12 (IL-12) level. The results by measuring cytokines in the aqueous humor samples shows that the IL-12 level in aqueous humor decreased with increasing severity of DR, and this correlation was significant (r=–0.482, p<0.001). Table 4 shows the sampling sizes for each group according to the ETDRS retinopathy severity scale.

According to the ETDRS retinopathy severity scale, 28 eyes were level 10, 23 eyes were level 20, 26 were level 35, 18 eyes were level 43, 13 eyes were level 47, eight eyes were level 53, seven eyes were level 65, eight eyes were level 75, and five eyes were level 81. Table 3 and Table 4 and Figure 1, Figure 2, Figure 3, Figure 4, Figure 5, Figure 6, Figure 7, and Figure 8 show the relationship between the concentrations of the assayed cytokines and DR severity. The aqueous humor levels of IL-1β, IL-6, IL-8, MCP-1, and IP-10 increased as DR increased; this correlation was significant. However, the concentration of VEGF was not correlated with DR severity. In addition, the aqueous humor levels of IL-10 and IL-12 decreased as DR severity increased; this negative correlation was significant.

**Table 3:** Relationship between the concentrations of the assayed cytokines and the severity of DR.

Level^a^	N	VEGF (SD)	IL-1β(SD)	IL-6(SD)	IL-8(SD)	MCP-1(SD)	IP-10(SD)
10	28	967.0(425.1)	10.0(12.4)	32.1(33.8)	22.8(22.1)	252.5(227.6)	2.1(1.73)
20	23	952.8(355.9)	11.0(18.4)	33.5(37.1)	20.6(25.5)	303.6(159.32)	2.5(2.7)
35	26	956.4(378.7)	9.2(14.4)	33.1(40.6)	22.7(31.1)	339.5(244.8)	5.6(4.0)
43	18	1084.7(349.9)	10.7(16.4)	33.2(43.3)	24.4(33.1)	468.8(273.9)	5.5(3.9)
47	13	1172.6(423.1)	18.8(15.3)	56.6(51.4)	29.2(33.7)	645.2(318.7)	9.5(12.1)
53	8	1177.3(534.5)	22.7(17.1)	106.7(52.7)	49.4(41.5)	921.2(391.1)	22.3(16.7)
65	7	1142.7(573.3)	23.7(29.0)	116.8(60.4)	51.0(22.8)	1215.1(435.9)	31.3(24.4)
75	8	1051.4(296.6)	27.6(36.0)	147.0(97.1)	75.7(58.9)	1286.6(383.4)	34.3(20.1)
81	5	1165.4(326.8)	45.8(33.1)	188.6(106.6)	74.4(59.3)	1630.8(601.2)	29.2(18.6)
P value^b^		0.733	0.003	<0.001	0.001	<0.001	<0.001

^a^ETDRS retinopathy severity ^b^Kruskal–Wallis test

**Table 4:** Relationship between the concentrations of the assayed cytokines and the severity of DR.

Level^a^	N	IL-10 (SD)	IL-12 (SD)
10	28	6.2(5.8)	16.2(11.4)
20	23	5.8(5.5)	13.7(8.9)
35	26	5.2(4.3)	11.5(9.0)
43	18	5.1(3.9)	7.3(5.8)
47	13	4.5(3.3)	6.3(5.3)
53	8	2.7(2.8)	4.0(2.8)
65	7	3.0(2.7)	4.4(3.5)
75	8	3.4(2.5)	4.3(2.6)
81	5	2.4(2.3)	3.0(2.5)
P value^b^		0.037	<0.001

^a^ETDRS retinopathy severity ^b^Kruskal–Wallis test

## Discussion

Many reports have shown that inflammation is an important factor that induces DR-related changes. Because it has been demonstrated that many cytokines and chemokines play important roles in DR, we must pay more attention to inflammation. Recent clinical and laboratory investigations have shown that diabetic subjects have an overall increased level of inflammatory activity relative to nondiabetic subjects [6-15]. Analyzing aqueous humor provides a useful tool for understanding the pathophysiology and treatment responses of many ocular conditions [26,30]. In our study, we simultaneously measured the concentrations of 27 human aqueous humor cytokines in samples from a large number of diabetic patients with or without retinopathy and nondiabetic controls. To our knowledge, this study is the first to analyze the levels of such a high number of cytokines in a comparatively large number of samples; in addition, this study included DR patients with different disease severities according to the ETDRS severity scale. The major findings of this study are as follows: (1) Compared with the nondiabetic controls, diabetic patients had significantly higher concentrations of IL-1β, IL-6, IL-8, MCP-1, IP-10, and VEGF and significantly lower concentrations of IL-10 and IL-12 in the aqueous humor. (2) The aqueous humor levels of IL-1β, IL-6, IL-8, MCP-1, and IP-10 are closely correlated with the severity of DR; however, the VEGF concentration was not correlated with the severity of DR. (3) The concentrations of IL-10 and IL-12 decreased as DR severity increased, and the negative correlation was significant.

IL-1β is a proinflammatory cytokine and an angiogenic mediator in several systems in diabetic patients, including the aqueous humor, vitreous, and tears [31-35]. Intravitreal injection of IL-1β accelerates the apoptosis of retinal capillary cells via the activation of nuclear factor kappa-light-chain-enhancer of activated B cells; this process is exacerbated under high-glucose conditions [36]. A previous study demonstrated that animals were protected from diabetes-induced retinal pathology by IL-1β receptor knockout [37]. In the current study, the IL-1β concentrations in the diabetic patients were significantly higher than the concentrations in the nondiabetic controls. This result also suggests that IL-1β may play a role in the development of retinopathy in patients with diabetes.

Studies of IL-6 concentrations in the aqueous humor, vitreous, tears, and serum of patients with DR have yielded contradictory conclusions [27,38,39]. In our study, the IL-6 levels in the diabetic patients were significantly higher than those in the nondiabetic controls. In addition, the IL-6 level was closely correlated with the severity of DR. IL-6 is a multifunctional cytokine that has proinflammatory and angiogenic functions through the induction of VEGF [12,40]. In addition, it has been reported that IL-6 is involved in the breakdown of the blood–retinal barrier [41]. In patients with DR, the level of inflammation gradually increases as the proliferative pathogenic process and neovascularization progress. Our results indicate that the more severe proliferative forms of DR involve serious blood–aqueous barrier dysfunctions due to the higher concentrations of IL-6.

IL-10, which is produced by monocytes and macrophages, is one of the main anti-inflammatory cytokines. IL-10 limits inflammation by reducing the synthesis of proinflammatory cytokines such as IL-1 and TNF-α, by suppressing cytokine receptor expression and by inhibiting receptor activation [42]. In addition, IL-10 prevents angiogenesis by downregulating VEGF expression [43]. IL-12 possesses antiangiogenic activity that is mediated by the stimulation of T-helper lymphocytes and the induction of IP-10 expression [44]. In accordance with previous results, the concentrations of IL-10 and IL-12 in the samples from the diabetic patients were significantly lower than the concentrations for the nondiabetic controls [8,22,23]. Our results suggest that low levels of circulating IL-10 (anti-inflammatory and antiangiogenic) and IL-12 (antiangiogenic) are involved in the pathogenesis of DR.

Chemokines are small heparin-binding proteins that bind to their cognate G-protein coupled receptors (GPCRs) to elicit cellular responses [45,46]. Based on cysteine residue positioning, chemokines are classified into four subfamilies: C, CC, CXC, and CX3C [45,46]. IL-8 and IP-10 are categorized as CXC chemokines, and MCP-1 is categorized as a CC chemokine. IL-8 is the major attractant and activator of neutrophils and T lymphocytes but not monocytes, and increased levels of IL-8 in proliferative diabetic retinopathy (PDR) are associated with a higher extent of large-vessel gliotic obliteration [45]. IP-10 selectively attracts activated T lymphocytes, the only inflammatory cells that express the chemokine receptor CXCR3 [46]. MCP-1 regulates the migration and infiltration of monocytes/macrophages via the chemokine receptor CCR2 but has no effect on neutrophils [47]. In accordance with previous results, the IL-8, IP-10, and MCP-1 concentrations in the samples from the diabetic patients were significantly higher than the concentrations for nondiabetic controls, and the aqueous humor levels of IL-8, IP-10, and MCP-1 were closely correlated with the severity of DR in our study [20,41,45,48]. All of the evidence indicates that inflammation is an important molecular mechanism in the development and progression of diabetic retinopathy and that the severity of ocular inflammation is closely correlated with the severity of DR [22,23,38].

VEGF is an endothelial cell mitogen that induces increases in vascular permeability and angiogenesis, enhances collateral vessel formation, and increases the permeability of the microvasculature [10]. The aqueous humor levels of VEGF have been found to be markedly increased in patients with DR, and the VEGF level has been reported to be significantly correlated with the severity of diabetic retinopathy [27]. In the current study, the concentrations of VEGF in the samples from the diabetic patients were significantly higher than the concentrations for the nondiabetic controls; however, VEGF was not correlated with the severity of DR (r=0.161, p=0.062). There may be several reasons for this difference. First, the severity of diabetic retinopathy is correlated with the severity of retinal neovascularization, which depends on VEGF. The concentration of VEGF has been found to be markedly increased in the vitreous of the eyes of patients with PDR; however, patients with severe PDR were relatively rare in our study. Second, although several previous studies have demonstrated that the concentrations of VEGF in the vitreous of patients with DR are significantly higher than the concentrations in nondiabetic controls, few studies have shown that the VEGF level in the aqueous humor is absolutely positively correlated with its level in the vitreous [49-52].

In recent years, intravitreal injection of different types of anti-VEGF has been used to inhibit VEGF’s angiogenic activity in managing PDR. The primary outcome measures for treatment with intravitreal injection of anti-VEGF include the regression rate of new vessels and the time to recurrence of new vessels. However, the effect of this treatment is not perfect, and the regression rate of new vessels following anti-VEGF treatment has been shown to range from 62% to 87.5%; the average time before retinal neovascularization recurs was found to range from 2 weeks to 3 months [53-57]. All the evidence has led us to think about additional mechanisms and new methods for treating DR. Our previous study demonstrated that the upregulation of MCP-1 begins in the early stage of DR and increases as the disease develops [58]. In addition, neuronal MCP-1 plays an important role in retinal microglial activation [58]. In our present study, the MCP-1 level was highly increased in DR patients and was highly correlated with the severity of DR (r=0.693, p<0.001). Based on the results of our study and others, we believe that detecting MCP-1 may be a useful method for determining the level of DR damage and that anti-MCP-1 treatment may be an effective way to treat DR.

The limitations of our study should be noted. First, the number of patients with severe PDR enrolled in the study was relatively low due to our comparative cross-sectional design. The association between cytokine levels and severe PDR must be studied further. Second, the concentrations of the cytokines in vitreous samples and serum were not determined. The cytokine levels in the vitreous are usually higher, and the analysis of vitreous would more accurately reflect the intraocular levels of cytokines and the status of the retina. In addition, an analysis of the cytokine levels in the serum would demonstrate whether the higher concentrations of cytokines in the aqueous humor are due to increases in the serum concentrations or due to the partial aggregation of cytokines in the aqueous humor and the breakdown of the blood–ocular barrier. The relationships among the cytokine levels in the aqueous humor, vitreous, and serum must be studied further.

In conclusion, the present study showed that the aqueous humor levels of IL-1β, IL-6, IL-8, MCP-1, IP-10, and VEGF were higher in patients with DR and that the levels of all of these cytokines, except VEGF, were closely correlated with the severity of DR. In addition, the aqueous humor levels of IL-10 and IL-12 were significantly lower in patients with DR, and the concentrations of these two cytokines decreased as the severity of DR increased. Various cytokines associated with inflammation and angiogenesis may contribute to the pathogenesis of DR, and chemokines, especially MCP-1, may be more closely related with disease development. Our study indicates that the intravitreal treatment of DR should be included in comprehensive DR treatment plans and that anti-inflammatory and antineovascularization agents should be used simultaneously.
